# BrainWiki—A Wiki-Style, User Driven, Comparative Brain Anatomy Tool

**DOI:** 10.3389/fnana.2020.548172

**Published:** 2020-10-19

**Authors:** Linda Forsell, Esther Naomi Vos, Keerthi Jayaraman, Axel Edman, S. Abid Hussaini

**Affiliations:** ^1^Department of Pathology and Cell Biology, Taub Institute, Columbia University Irving Medical Center, New York, NY, United States; ^2^Karolinska Institutet, Stockholm, Sweden

**Keywords:** brain anatomy, brain atlas, comparative anatomy, mouse brain, human brain, brain circuits, brain pathology, brain functions

## Abstract

The mouse is the most important animal model within neuroscientific research, a position strengthened by the wide-spread use of transgenic mouse models. Discoveries in animals are followed by corroboration in humans, and the interchange between these fields of research is essential to our understanding of the human brain. With the advent of advanced technologies such as single-cell transcriptomics, epigenetic profiling and diffusion MRI, many prominent research institutes and collaborations have emerged, aiming to construct complete human or mouse brain atlases with data on gene expression, connectivity and cell types. These initiatives are indispensable resources, but frequently require extensive, time-consuming development, and rely on updates by the provider. They often come in the shape of applications which require practice or prior technical know-how. Importantly, none of them place the human and the mouse brain next to each other to allow for immediate comparison. We present BrainWiki, a user-friendly, web-based atlas that links the human and the mouse brain together, side-by-side. The platform gives the user a simple overview of brain anatomy along with published articles relating to each brain region that allows the user to delve deeper into the current state of research concerning circuitry, brain functions and pathology. The website relies on interactivity and supports user contributions resulting in a dynamic website that evolves at the pace of neuroscience. It is designed to allow for constant updates and new features in the future which will contain data such as gene expression and neuronal cell types.

## Introduction

To understand the function of the brain and its diseases, scientists often compare brain regions across different species. There have been major advances in technologies to understand the human brain, but all clinical testing is preceded by animal testing. Therefore, the use of animals in understanding human diseases is inevitable. Although the human brain is highly homologous to the primate brain, most of the neuroscience research is done in rodents. In fact, rodents are the most commonly used animals in biomedical research with about 40% of neuroscience articles (between 2000 and 2004) based on research performed on rats and mice (Manger et al., 2008; Ellenbroek and Youn, 2016). In 2017 alone, the National Institute of Health (NIH) spent 7.317 billion USD on neuroscientific research and a substantial proportion of this funding was allocated to research in mice (Pankevich et al., 2012; National Institutes of Health, 2017). The common house mouse (*Mus musculus*) shares 97% genetic homology with humans and has become an indispensable tool for research with the rapid development of genetic engineering tools including the CRISPR/Cas9 system leading to transgenic and knock-in mice. Progress made in biomedical research to understand the mechanism of Alzheimer’s disease, Huntington’s disease and Parkinson’s disease, and preclinical trials are strongly dependent on mice (Rockenstein et al., 2007; Fisher and Bannerman, 2019). A considerable amount of homology exists between the human and the mouse brains too, and the field of comparative neuroanatomy has long explored the correlations and differences between them. Many brain functions can be attributed to the homologous brain regions in the human and the mouse brain. However, for translational research, any findings in the mouse brain must always be corroborated in the human brain to assist further research and clinical implementation (Morrissette et al., 2009). This calls for a simple tool that allows frequent comparison and integration of data from researchers of mouse and human brains.

There are several promising projects with vast amounts of data pertaining to anatomy, morphology, physiology and connectivity of the brain in different species that have been the foundation of neuroscientific research and are cornerstones in research development. But several obstacles have to be addressed to fully benefit from these advancements. Challenges outlined by the BRAIN Initiative include finding solutions to automate the collection of the immense amounts of data, finding a common classification for neurons (Bota and Swanson, 2007) and integrating these into a comprehensible cell atlas (Ecker et al., 2017). The Allen Brain Atlas,^1^ created by the Allen Institute for Brain Science, is possibly the most established of these, providing many web-based datasets including: 3D atlases of gene expression in the mouse, macaque and human, a mouse connectivity atlas, references atlases for both mouse and human and targeted human gene expression studies exploring subjects like schizophrenia and aging. These datasets and web applications are open source and intended as a resource for neuroscientists (Jones et al., 2009), as well as attempting to set a standardized data format to allow integration of data from throughout the research community (Kuan et al., 2015). The Allen Brain Atlas has been used extensively within research, industry and academia alike, and is considered one of the chief contributions to the field (Jones et al., 2009; Gilbert, 2018). Another project of note is The Human Connectome Project^2^ (Van Essen et al., 2012), an NIH funded, 40 million USD project initiated in 2009, using advanced imaging techniques to construct a map of structural and function human brain connections. Other noteworthy pursuits include the Human Brain Project,^3^ a European project which aims to build a simulation of the human brain (Hampton, 2014); the Swiss Blue Brain Project^4^ attempts to create a digital reconstruction of the rodent and ultimately the human brain (Markram, 2006); and the Brainnetome,^5^ a Chinese initiative, aims to create an *in vivo* map of functional brain regions in human and non-human primates (Fan et al., 2016; Nowinski, 2017).

Although digitalized, many of the above projects are still static and will rely on updates by the provider. They often come in the shape of complex applications which also allow for data input and processing. As an example, the Workbench application provided by the Human Connectome Project, requires downloading of their application, and for anyone without a research technician’s skills, it could take several hours to understand and navigate basic functions. The application is no doubt of substantial benefit for those requiring advanced processing and detailed data acquisition, but for a broader base of scientists with vastly varying levels of technical knowledge, it does not provide fast access to general information. Some projects like the BRAIN initiative aim to release their atlas when it is deemed complete, a prospect which will require time, and may prove uncertain in terms of results. None of these initiatives offer a side-by-side juxtapositioning of the human and rodent brain, possibly due to the fact that the exact correlation between brains in different species is still unknown, and because the brains simply do not match completely. Consequently, none of these initiatives have created a platform to compare the human and mouse brain and allow simple extraction of the information the databases contain. Scientists, clinicians, students and others working in different parts of the broad field of neuroscience, lack a simple tool for putting together their in-depth knowledge about a specific area of research into the greater perspective of the entire brain and compare them across different species.

**FIGURE 1:**
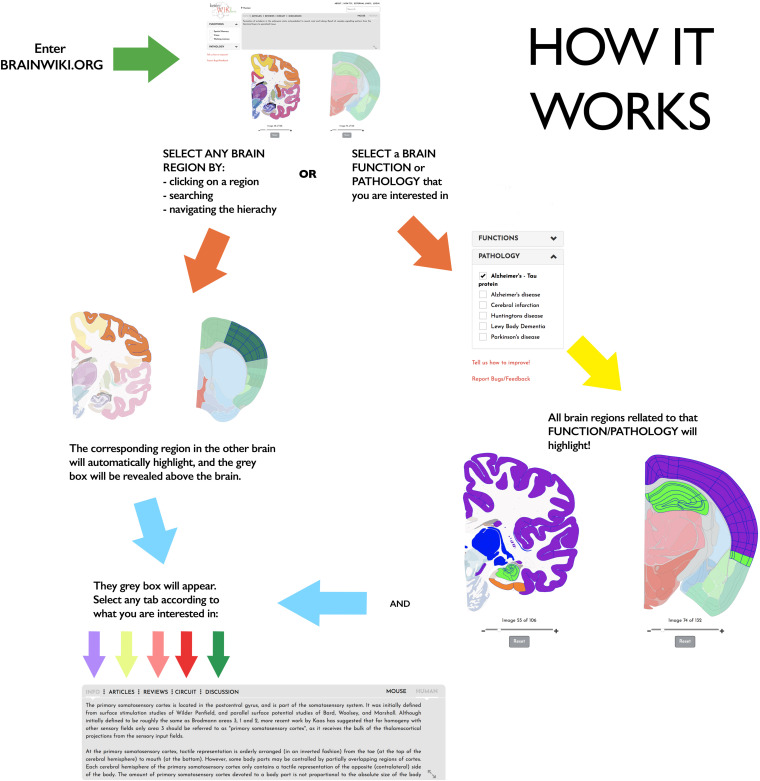
How BrainWiki works. The figure explains how to enter and navigate BrainWiki. After entering Brainwiki.org, the user can navigate the mouse or the human brain region by clicking on a brain region, by searching for a brain region, or using the hierarchy at the top. The corresponding brain region in the other brain will automatically highlight as well, and the gray box will appear. User can select any tab in the gray box to find relevant information of interest. The user may also choose to select a brain function or a pathology from the menu on the left. This will highlight all brain regions related to that brain function or disease in both brains, and a similar gray box will appear.

To bridge this gap, we came up with a comparative anatomy tool, BrainWiki that places the human and the mouse brain atlas side-by-side, allowing direct comparison of the two neurological systems (Figure 1). It aims to give the user easy access to information about scientifically relevant data pertaining to neuroscience, without the need for previous technical know-how, and is available on a user-friendly website which aims to always stay updated through the input from users around the world. This will be accomplished by allowing users to communicate and add information to any part of the mouse or human brain, a concept that has proven successful and reliable on many other wiki-pages, Wikipedia being the most famous. BrainWiki will also provide links to more detailed and advanced applications and data processing tools. The platform is based on the Allen Institute for Brain Science’s reference atlases and is therefore aligned with their common coordinate framework. This is an invaluable resource although in the original form the Allen brain atlases lack possibilities for direct user interactivity and cross-comparison of the human and mouse brain. By always allowing for updates as researchers gain more knowledge of the brain, the BrainWiki concept does not have to be conclusive nor entirely accurate at launch. It can display the correlations that do exist and explain the differences that cannot be compared between species. The website is intended to further research and allow researchers to extrapolate their specific knowledge into an understanding of the greater picture, and to inspire and give the user perspective outside their own field of research.

## Results

**FIGURE 2:**
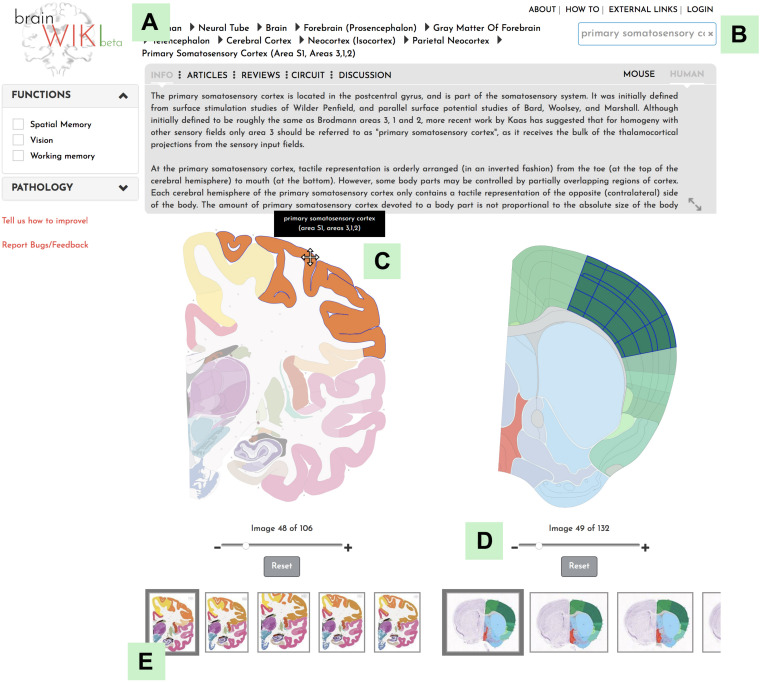
BrainWiki features. **(A)** The hierarchical navigation menu **(B)** The search tool **(C)** Hovering on the brain pops up names of the brain regions **(D)** The zoom tool **(E)** The thumbnails allow different sections/slices of the brain to be selected.

**FIGURE 3:**
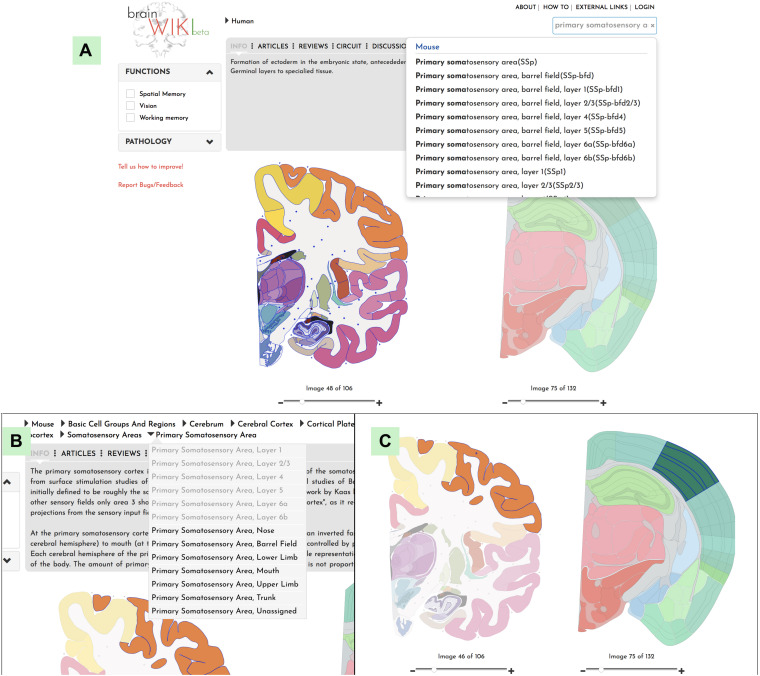
Navigation. Explanation of how to navigate to a desired brain region in BrainWiki. **(A)** Search function in the upper right corner allows the user to search for a region of interest. **(B)** Hierarchal navigation of the brains by a dropdown menu, can be found in the top bar. This is useful for anyone familiar with gross brain anatomy. If you have currently selected for example the Primary Somatosensory area of the mouse brain, the menu above will show Mouse > Basic Cell Groups and Regions > Cerebrum > Cerebral Cortex > Isocortex > Somatosensory Area > Primary Somatosensory Area, according to the hierarchy of the mouse brain. Each level of the hierarchy can be revealed and expanded by clicking the arrows in between. Simply select any region in the human brain and the hierarchy will swap over to the human brain. **(C)** Finally, a click on any region of the human brain will highlight it, together with the corresponding mouse region.

BrainWiki^6^ is designed to be a simple, clutter-free visual tool that is easily accessible on the web without any installation. Along with the pictorial representation of how BrainWiki works (Figure 1), we provide a step-by-step explanation of each feature in BrainWiki (Supplementary Video 1). The user interface or the front end displays a human brain and a mouse brain side by side, allowing for a quick and simple overview of how human brain regions correspond to mouse brain regions according to current anatomical knowledge. The brain regions can be navigated by using the hierarchical menu (Figure 2A), by entering a region name in the search tool (Figure 2B) or by simply clicking anywhere on the brains themselves (Figure 2C). Hovering the mouse pointer on the brain displays the region names as pop-ups, allowing a quick way to browse the brain. When a region in one brain is selected, the corresponding region in the other brain highlights automatically. The hierarchical menu (Figure 2A) displays the current selection and its parent regions. It gives a clear, tree-like overview and useful for quick navigation if one is familiar with the gross anatomy of the brain. The search field (Figure 2B) allows jumping quickly to a brain region by typing names or abbreviations. It autosuggests a drop-down list of brain regions that can be selected and navigated to. The brains can be moved around and can be zoomed in/out by using the mouse wheel or by using the dedicated zoom tool (Figure 2D). The entire brain can be visualized by navigating the thumbnails of sections below each brain (Figure 2E). The human brain has 106 sagittal sections and the mouse brain has 132 coronal sections. The selections will stay highlighted even when moving to a different slice. Examples of hierarchical menu, search tool and clicking to select a brain region is shown in Figure 3.

**FIGURE 4:**
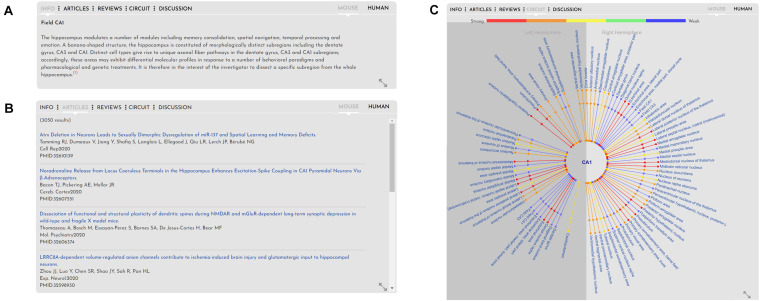
Gray box. Figure explaining the information in the gray box pertaining to the brain regions, pathologies and functions. From left to right the tabs in the gray box show **(A)** Info: shows any relevant information available about the brain region (or function or pathology, if those are selected). **(B)** Articles: show published articles pertaining to the brain region (CA1) sorted by most recent. Articles tab, if selected, would show recent and relevant reviews pertaining to the brain region (not shown). **(C)** Circuits: shows connections to and from the selected brain region via simple lines (for details, see Figure 5). Discussion tab shows a forum for users to discuss brain anatomy, function and pathology (not shown).

After selecting a brain region, an expandable gray box opens above the brains. The box contains tabs with different types of content (Figure 4). These include: Information, Articles, Reviews, Circuit and Discussion tabs. In the far-right corner of the box the user can toggle between the mouse and the human brain.

The information tab contains material gathered from sources like Wikipedia, Encyclopedia Britannica and a multitude of scientific articles. For example, in the box pertaining to field CA1, it states that *“The hippocampus modulates a number of modules including memory consolidation, spatial navigation, temporal processing and emotion. A banana-shaped structure, the hippocampus is constituted of morphologically distinct subregions including the dentate gyrus, CA3 and CA1….”* This text, as well as data in the other tabs, can and will be modified by the users themselves to add increasing detail to the content and to include new findings in the rapidly evolving field. References for each individual addition are marked in a standard way by superscripts, and the corresponding citation is found in the footnotes below the text.

Articles and reviews have been imported to brain regions by embedding specified PubMed searches into these respective tabs. The Articles tab is filtered to include a broad selection of published material pertaining to the selected brain region, showing the most recent articles first, and divided into research performed on human or mouse.

Reviews, on the other hand, have been added to narrow the search results by including additional search words, restricting the search to only the most recent 5 or 10 years, filtering for reviews, and separated by human or rodent.

**FIGURE 5:**
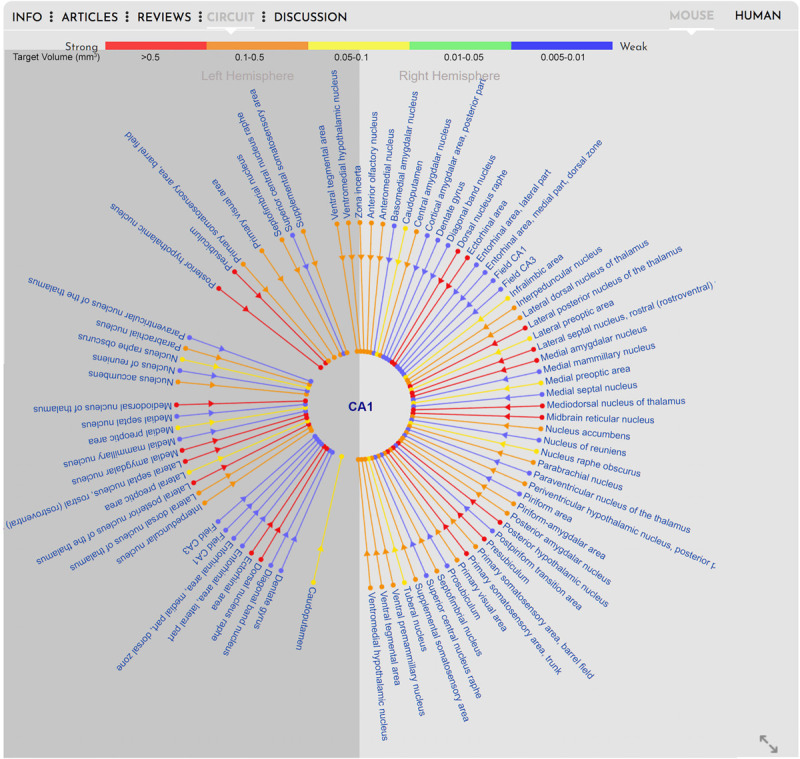
Circuitry. Visualization of the circuitry interface embedded in the gray box of BrainWiki brain regions. The selected brain region, CA1 in the mouse, is shown in the middle, and known connections to and from the CA1 are connected via simple lines to the center. Different colors signify the relative strength of the connections, arrows on the lines show afferent vs. efferent connections, and a division of the brain hemispheres identify connections on the same (right) side vs. connection traversing to the opposite (left) side of midline.

In contrast to showing standard cross-species anatomy, representation of brain circuitry has proven a more challenging task, due to the ongoing challenge of acquiring research data on human tractography. The tractography data provided for the human brain on BrainWiki.org is therefore nascent in its nature, and expected to develop and advance rapidly in the forthcoming years through projects such as the Human Connectome Project as well as advancements in pre-existing and new technology, such as diffusion MRI (Van Essen et al., 2012; Yeh et al., 2018). Despite the gaps in data, we designed a forward-thinking framework capable of incorporating upcoming circuitry studies into a cohesive visualization. To display brain connectivity in an easy-to-read visualization, we used a radial diagram centered around the currently selected brain region, i.e., CA1 (Figure 5). Each connected brain region is displayed in a radial fashion around the selected target region. Each connecting line indicates afferent and/or efferent connections with arrows and is colored by the relative strength of the connection. Finally, the radial diagram is divided into two halves: lines extending to the right of center represent connections to/from the ipsilateral (same) hemisphere, and lines on the left to/from the contralateral (opposite) hemisphere.

Unlike the human brain, the connectivity in the mouse brain is well documented because of the neuroanatomical tracing data that has been made available by the Allen Brain Institute via their Mouse Connectivity database^7^ (Oh et al., 2014). This database was generated by injecting viral tracers into specific brain regions and tracing and quantifying the connectivity with serial 2-photon tomography. Using this connectivity database, we have automatically generated radial diagrams that displays connectivity between mouse brain regions. To keep the circuit simple, for any selected brain region, we display only incoming projections from other regions. For example, in Figure 5, we show projections to CA1 from ipsilateral brain regions (right) and from contralateral brain regions (left). The color scale depicting the strength of the connections is derived from the database. The colors red, orange, yellow, green and blue indicate target volume (in mm^3^) 0.005–0.01, 0.01–0.05, 0.05–0.1, 0.1–0.5, and > 0.5, respectively. Because of the vast number of connections that are possible for some brain regions, we only display the top 50 connections based on the strength of the connections.

**FIGURE 6:**
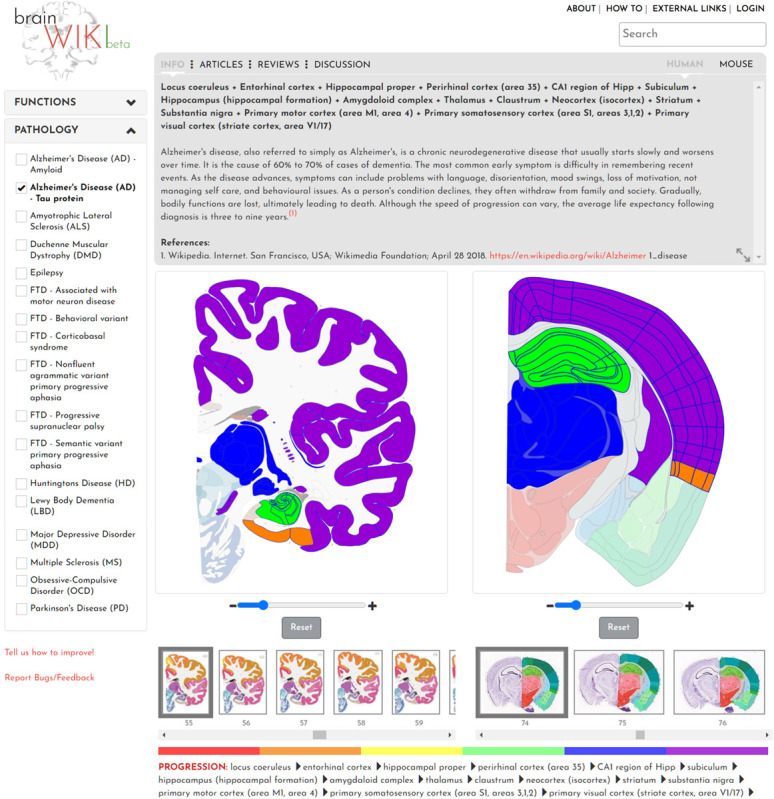
Pathologies and functions. Figure showing the interface of functions and pathologies through the example of Alzheimer’s disease Tau protein progression. Here, the progression of Alzheimer’s is visualized through the color of the highlighted brain regions, beginning with red and reaching purple at a late stage of the disease. A color bar below the brains explains the order in which it evolves throughout the brain. The gray box above shows an explanatory text about a pathology.

In addition to regional and connectivity mapping, the interface also includes functional and disease annotations. In the left-hand panel, two key features display various Functions and Pathologies (Figure 6). Selecting one of the checkboxes in either utility will highlight all brain regions involved in that certain brain function or pathology, opening up a gray box with information and data specific to the selection. When clicking on any of the functions, all brain regions pertaining to that function will be highlighted. However, since not all regions will be visible in one brain slice, the info tab lists all the regions involved in the function. Similarly, for pathology, all brain regions affected by the disease will be listed on the info tab (Figure 6, Info tab). For both function and pathology, the information will evolve quickly, and we will make every effort to keep it updated. As an example, when adding Alzheimer’s disease to the website, the identification of the current state of research with regards to pathology and the progression of the disease throughout the brain was attempted. Alzheimer’s pathophysiological theory explores two major fields, the accumulation of amyloid beta protein in the brain, and abnormal phosphorylation followed by defective degradation of Tau protein leading to aggregation of neurofibrillary tangles. Hence, in BrainWiki, two different selections were created to separate these two pathways.

As a further example, in the Tau pathway, current research has revealed the locus coeruleus as the first point of decreased Tau degradation, followed by a stage involving the entorhinal cortex (Braak et al., 2011). This phase is followed by engagement of CA1, primarily the Stratum Pyramidale, and thereafter the rest of the Hippocampus. A limbic stage ensues, involving the Subiculum and the rest of the hippocampal formation, followed by the Amygdala, Thalamus and Claustrum. After this phase tauopathy involves extensive parts of the Isocortex including the primary sensory, motor and visual areas. Finally, the Striatum and Substantia Nigra are affected (Serrano-Pozo et al., 2011; Korolev, 2014; Kumar et al., 2015). This progression is visualized in BrainWiki by highlighting all these brain regions on the respective atlases in six different colors. The colors follow a gradient, with red colors denoting regions engaged at the primary stage, and purple denoting the last stage. A legend for how to interpret the colors is found below the brains. The progression is also explained in text in the gray box, and the brain regions are spelled out below the brains. A third progression path for Alzheimer’s disease has been described following the brain regions that appear to have the largest effect on symptoms. This path appears to follow the Tau protein progression more closely that the Amyloid path and is in general less precisely described but may be of interest as a future addition to the site.

The discussion tab in the gray box uses Disqus, a discussion platform which is widely used by several communities for exchanging ideas. It also has wide social networking capabilities such as twitter, facebook, etc. for quick sharing of information. Every brain region, pathology or function comes with its own unique web link and therefore can be discussed and commented upon directly. For example, if a user needs help with references for the function Olfaction, then by simply sharing the following link: https://www.brainwiki.org/index.php?t=function&b=olfaction, the user can engage in discussion or feedback.

All information on BrainWiki is far from definitive and the data added at launch represents a foundation to be expanded on. Therefore, in addition to the front end, an intuitive back end has been developed to allow interaction and continuous updates by the users, who are given access to edit most of the content on the site. This approach ensures that the site stays relevant and reflects current knowledge within the field.

**FIGURE 7:**
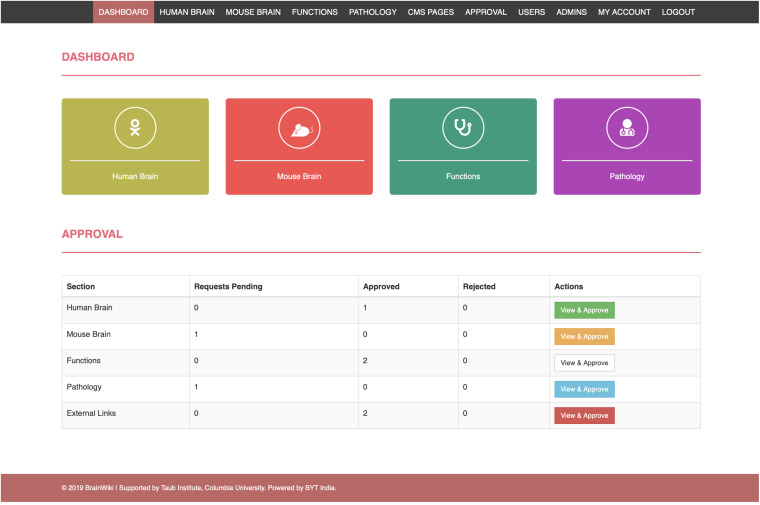
The back end of BrainWiki. The back end is a portal for users, moderators and administrators to edit and manage the content on BrainWiki. Moderators and administrators have additional roles such as allowing user access, approving or rejecting user edits etc.

**FIGURE 8:**
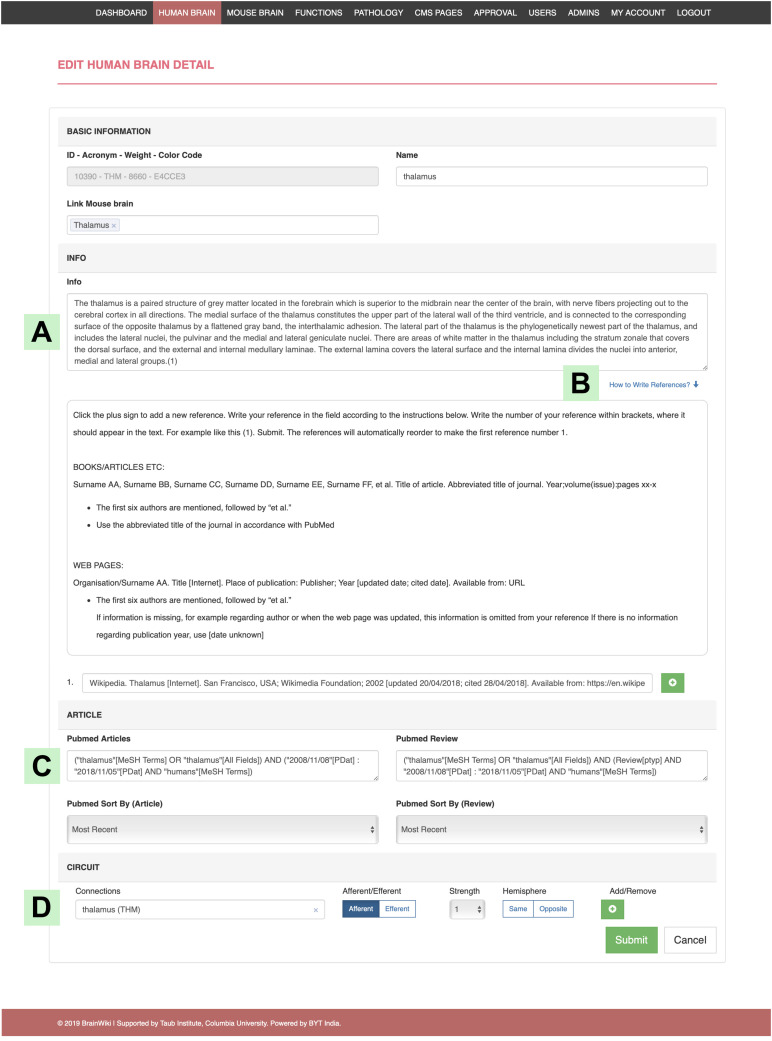
Editing in the back end. An example of the structure of the back end, showing the human Thalamus editing platform. **(A)** The field where you add/edit text in the information tab of the gray box. **(B)** Click on “How to Write References” for instructions on references. **(C)** This field allows you to add search details from PubMed, which will automatically embed the search into the gray box in the front end. **(D)** Where to add/edit brain regions connected to the selected brain region, in this case the Thalamus. This function allows you to set if the connection afferent/efferent or both, its strength compared to other connections and if the connection travels to the opposite hemisphere or not.

By creating an account on BrainWiki, users are granted access to make contributions. On logging in, the back end menu allows the users to choose what to edit (Figure 7). To edit specific brain regions, select human or mouse, then search the website for your region of interest, and click edit to the right. Information can be added in the text box (Figure 8A) along with references. Instructions on how to add or remove references including formatting details are explained (Figure 8B). Once the references are added, the reference numbers should be typed in-line within brackets for it to appear at the end of the page. The site automatically reorders reference numbers to follow the order of the text. Articles and reviews (Figure 8C) are embedded by performing a search on PubMed and adding the search details to the back end of BrainWiki. Circuit information can be edited (Figure 8D) by adding brain regions, by assigning if connections are afferent/efferent, their strengths and same/opposite hemisphere laterization. For detailed explanations on how to add or edit in the back end, several how-to videos are available in a link at the top right of BrainWiki.

When adding a function or pathology, the user will be requested to add brain regions involved in that feature: i.e., dysfunctional brain regions affected by the disease, or brain regions active in a specific brain function. For pathologies user will be asked to order the regions according to the disease progression in the brain. The slice number to be displayed in the front end, for both the human and the mouse brain, can be manually adjusted. There are also options to add information and articles similar to that for the brain regions.

BrainWiki will be moderated for accuracy. New or edited content will be reviewed by a team of brain anatomy experts who will confirm that the submissions contain relevant information and references. As a result, new modifications have been programmed to not appear on the site instantaneously after a user contribution. When a user edits the content and submits a change, the BrainWiki team administrator is notified about the pending request in the approval section on the back end (Figure 7). One of the experts reviews the content and notifies the administrator to either reject or approve the request. If rejected, the user is notified and given the opportunity to make edits and submit the content again. If the request is approved, the content is immediately pushed to the front end and will appear online. We will not judge the quality of the studies or references that comments refer to, and disputes/competing theories will be displayed side by side as such.

## Discussion

We have created a tool that we believe would fill an important gap in the field of comparative neuroanatomy by allowing users to directly compare two most commonly used species in neuroscience. We are fully aware that the mouse and the human brain cannot be fully matched, but the strength of BrainWiki is to reveal the similarities and differences in these brains. The hope is that the commonalities and differences in the brain anatomy and connectivity in the two brains will allow better understanding of our research problems.

The target audience for BrainWiki will include neuroscientific researchers, clinicians and students within subjects such as medicine, biomedicine, and neurology. Users are not required to download an application, nor any technical know-how to navigate BrainWiki. The interface is constructed in line with current, modern website navigation standards and therefore requires only common computer skills. This also applies to adding and editing in the back end.

BrainWiki is designed primarily for the needs of neuroscientific researchers and clinicians. These two groups will likely have overlapping but slightly different approaches to the site. Both will easily navigate to their purposes. As an example, a clinician researching Alzheimer’s disease in humans may discover a brain region which is affected early on in the disease. To determine whether the molecular pathophysiology of this region can be studied in mice, a simple click will allow her to find out if there is a corresponding mouse region and what research has been done on that region in animal models. Conversely, a pre-clinical researcher working on describing cells involved in spatial memory in the mouse, may be interested in knowing the involvement of the examined regions in various diseases. By clicking “Alzheimer’s disease—Tau protein” the brains will quickly display which regions are affected by that disease, and the progression pathway this disease takes throughout the brain, based on a color code explained below the brains. Table 1 shows suggested ways to utilize BrainWiki for different users, divided into these two different target groups.

**TABLE 1:** Utilization of BrainWiki.

**Suggested areas of use**		
**Clinician**	**Researcher**	**Both**
• Find mouse regions corresponding to a specific human region. • Discover research made on a specific brain region, and the corresponding research been made on animal models pertaining to the corresponding region. • See disease progression for neurodegenerative diseases and discover corresponding brain regions in mouse. • See circuits connected to a specific brain region, and toggle to view the similarities/differences of the corresponding mouse region circuits.	• Find human regions corresponding to a specific mouse region. • Discover research made on a specific brain region, and the corresponding research been made on humans pertaining to the corresponding region. • See circuits connected to a specific brain region, and toggle to view the similarities/differences of the corresponding mouse region circuits.	• Learn more about any specific brain region. • See disease progression for neurodegenerative diseases, and brain regions related to it. • Discuss with other researchers. • Discover regions involved in a specific function, and the corresponding mouse region. • Discover the different neuronal cell types within a region. • Discover evolutionary relationship between the two brains with respect to structure and function.
**Future usage**		
• Discover gene/protein expression in a specific human brain region. • Evaluate if same gene/protein is expressed in a corresponding mouse region. • Find other brain regions expressing a certain gene/protein. • Add a new brain region to the human brain. • Find stereotactic coordinates for specific neuronal cell types.	• Discover gene/protein expression in a specific mouse brain region. • Evaluate if same gene/protein is expressed in the human corresponding region. • Find other brain regions expressing a certain gene/protein. • Add a new brain region to the mouse brain. • Find stereotactic coordinates for specific neuronal cell types.	

*Suggested areas of usage of BrainWiki for clinicians and researchers.*

BrainWiki does not aim to be conclusive. It is a tool where crowd-sourced knowledge can be shared and gaps can be brought to the attention of the research community, with disagreements discussed and decision-making evolved. Akin to Wikipedia, the content on BrainWiki will be added and improved continuously by the users themselves. The data and construction at launch is a solid foundation to expand from, but several areas of improvement can be readily identified, and new features will be released periodically. An early goal is to add a feature describing gene and protein expression in different brain regions. For example, it could aid a researcher studying a novel potential therapy for Parkinson’s disease, who is interested in evaluating the drug in mice ahead of clinical trials. To determine the relevance of this, it would be of interest to know if the target protein of the drug is also expressed in the human brain region of interest. First the researcher would want to find the corresponding human region, which is immediately displayed after selecting the mouse brain region of interest. Thereafter, the researcher will easily find verification that the target protein exists in the selected human region by selecting the protein expression tab. In addition, a future function will display layers on the actual brain map, to be toggled on and off for display of data such as the expression of specific genes. When such a feature is toggled on, the selected gene would be visually displayed over the brain regions containing it. Another new feature will allow the user to add additional brain regions/points of interest. The site has been designed to allow for simple development in these directions. To evaluate our next steps of development, BrainWiki will host a poll to allow users to vote for the features that would serve them the best. See Table 2 for a list of proposed features to be added.

**TABLE 2:** Future of BrainWiki.

**Suggested features to be added**
• Gene/Protein expression
• Neuronal cell types
• Stereotactic coordinates
• Radiology visualization
• Additional reference brains
• Brain nuclei
• 3D visualization of brains

BrainWiki is built on a foundation which requires collaboration and will aim to further the work of others and link together preexisting data and tools. Compared to other neuro-mapping initiatives, BrainWiki does not purport to offer the most advanced platform for data processing and gathering; there are other platforms such as The Human Connectome application “Workbench” or the Whole Brain software described in the introduction, that are superior for these purposes. BrainWiki is instead intended as a hub which encourages users to link and refer to relevant pages providing such resources. The design of BrainWiki is user-friendly, intuitive, and the direct interactivity with users will make sure that the site stays relevant as they add and edit the content. But this makes BrainWiki reliant on a strong user base and their investment in the website as a tool—without user contributions the site will stagnate. The challenge is therefore to spread the word and engage researchers globally. By doing this, BrainWiki could become a platform for reliable neuroscientific information which can grow, expand and develop, as well as provide information about the most reliable tools for further data processing and interpretation provided by other entities.

## Methods

The foundation of BrainWiki are the Allen Human and Mouse brain Reference Atlases: “Human, 34 years, Cortex—Modified Brodmann” and “Mouse, P56, Coronal,” whose open source API (Allen Institute for Brain Science, 2015) has been modified to serve our purpose. The Human brain is sagittal reference atlas divided into 106 and the Mouse brain is coronal reference atlas divided into 132 brain section SVG (scalable vector graphics) images, respectively, which were initially downloaded and stored on our local server. Downloading the API, data and images, onto BrainWiki’s own server results in a faster website with shorter loading times and secures the functionality of our website in the event that the Allen Institute for Brain Science were to make incompatible modifications on their end. The atlas drawings and ontologies were downloaded from here: http://help.brain-map.org/display/api/Atlas+Drawings+and+Ontologies. We followed detailed examples outlined there which yielded high quality images. We used the SVG download service which returned the annotations of a specific image as SVG. The following link provides detailed examples on downloading SVGs: http://help.brain-map.org/display/api/Downloading+and+Displaying+SVG. Using this fetch service we retrieved data for both brains and built atlases resembling those on Allen Brain.

The utilization of the Allen Brain Atlas neuroscientific platform allows for simple future adaptations of the atlas content and collaboration with other tools using the same API, including data from Allen Brain Atlas itself which currently covers features for gene expression in the mouse and human brain, neuronal cell type electrophysiology and morphology, and mouse brain tractography (Stone et al., 2011).

At the outset, the website was designed aiming toward a user-friendly experience and targeting the inclusion of imperative neuroscientific data. With these criteria in mind, the site was programmed using standard HTML and CSS by the website developers who were also consulted to achieve a fast and fluid interface. To enable the content of the gray box to be updated when switching tabs without loading a different or new page, JavaScript AJAX language was applied. Primary tabs at launch include general information about the selected region, as well as tabs for articles and circuitry. Basic content derived from scientific articles and reliable online sources was added to the info tab, functions and pathologies by the BrainWiki team.^8^ This makes BrainWiki useful to users from launch and serves as a foundation for researchers and clinicians with more specific knowledge to expand upon. References to the content can be found at the bottom of the info tab. Articles and reviews have been imported to brain regions from PubMed’s open source API^9^ (Sayers, 2010). The data for Circuit for the mouse brain was generated automatically using Allen Brain Institute’s Mouse Connectivity database.^10^ We used the projection dataset^11^ and used the connectivity API^12^ to perform a target search on all possible brain regions and filter results to include only C57BL/6J mice. Further, we used 5 target volume ranges (Figure 5) to quantify our connectivity strengths. We then programmed the radial diagram to include only top 50 brain regions on each right (ipsilateral) and left (contralateral) hemispheres. For building the discussion platform, Disqus API^13^ was used which allows both registered users or guests to comment on a specific topic on BrainWiki.

In addition to the front end of the website, a user-friendly back end was devised to allow content to be added, edited, curated by users. The back end includes a feature to link brain regions constructed with PHP scripting language fetching the brain regions from the API, and the language-independent JSON format to transmit. Corresponding brain regions were initially identified through basic research performed by the team itself. Information was mainly obtained from Wikipedia, braininfo.org—a website provided by the University of Washington, Seattle, containing information on neuroanatomical structure and the Allen Brain atlas, with complementation from specific scientific articles.

The brain regions were then linked together at the website, followed by corroboration and editing of the anatomical links by some of the foremost researchers in human and mouse anatomy. The linking can be easily adapted when future research reveals flaws in the content.

**FIGURE 9:**
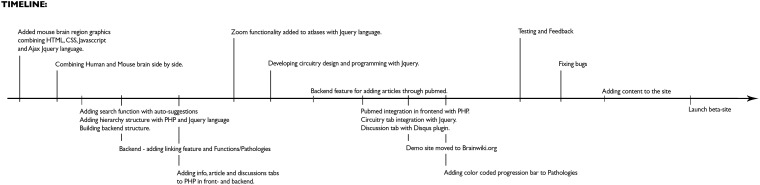
Development of BrainWiki. The chronological order of how BrainWiki was developed with technical details of features added until launch of beta-site.

Figure 9 for a timeline of the technical development of BrainWiki.

## Data Availability Statement

All datasets presented in this study are included in the article/Supplementary Material.

## Author Contributions

SH supplied the initial idea, and together with LF worked with concept development. LF created and designed the website, including user-experience and logotype aspects. SH and LF developed the framework for the content. EV, LF, and AE added initial data, including researching pathologies and functions, information about brain regions, functions and pathologies, and PubMed article searches. LF and KJ researched and connected linking brain regions. LF and SH wrote the manuscript. The website was programmed by the developers at BYT India. All authors contributed to the article and approved the submitted version.

## Conflict of Interest

The authors declare that the research was conducted in the absence of any commercial or financial relationships that could be construed as a potential conflict of interest.

## Footnotes

1 portal.brain-map.org

2 www.humanconnectomeproject.org

3 www.humanbrainproject.eu

4 bluebrain.epfl.ch

5 atlas.brainnetome.org

6 brainwiki.org

7 http://connectivity.brain-map.org/

8 brainwiki.org/about-us.php

9 https://www.ncbi.nlm.nih.gov/home/develop/api/

10 http://connectivity.brain-map.org/

11 http://help.brain-map.org/display/mouseconnectivity/Projection

12 http://help.brain-map.org/display/mouseconnectivity/API

13 https://disqus.com/api/docs/

## Supplementary Material

Supplementary Video 1 How to use BrainWiki. A quick-start video tutorial explaining the core features of BrainWiki. It shows how to navigate through the website and what each feature does. Some important highlights are: 10 s: Shows the core feature of automatic linking of brains. 30 s: Shows three ways to navigate brain regions; Clicking, Searching or Hierarchy. 1 min 19 s: shows brain slices. 1 min 39 s: shows the various tabs within the gray box. 2 min 07 s: shows the simplistic circuit diagram used for brain connectivity. 2 min 50 s: explains how pathology is visualized. Video explanations also available at brainwiki.org/howto.php.
